# Automatic recognition of uterine contractions with electrohysterogram signals based on the zero-crossing rate

**DOI:** 10.1038/s41598-021-81492-1

**Published:** 2021-01-21

**Authors:** Xiaoxiao Song, Xiangyun Qiao, Dongmei Hao, Lin Yang, Xiya Zhou, Yuhang Xu, Dingchang Zheng

**Affiliations:** 1 Faculty of Environment and Life, Beijing University of Technology, Beijing International Science and Technology Cooperation Base for Intelligent Physiological Measurement and Clinical Transformation, Beijing, 100124 China; 2 Department of Obstetrics, Peking Union Medical College Hospital, Beijing, 100730 China; 3 Centre for Intelligent Healthcare, Faculty of Health and Life Science, Coventry University, Priory Street, Coventry, CV1 5FB UK

**Keywords:** Data processing, Disease prevention

## Abstract

Uterine contraction (UC) is an essential clinical indicator in the progress of labour and delivery. Electrohysterogram (EHG) signals recorded on the abdomen of pregnant women reflect the uterine electrical activity. This study proposes a novel algorithm for automatic recognition of UCs with EHG signals to improve the accuracy of detecting UCs. EHG signals by electrodes, the tension of the abdominal wall by tocodynamometry (TOCO) and maternal perception were recorded simultaneously in 54 pregnant women. The zero-crossing rate (ZCR) of the EHG signal and its power were calculated to modulate the raw EHG signal and highlight the EHG bursts. Then the envelope was extracted from the modulated EHG for UC recognition. Besides, UC was also detected by the conventional TOCO signal. Taking maternal perception as a reference, the UCs recognized by EHG and TOCO were evaluated with the sensitivity, positive predictive value (PPV), and UC parameters. The results show that the sensitivity and PPV are 87.8% and 93.18% for EHG, and 84.04% and 90.89% for TOCO. EHG detected a larger number of UCs than TOCO, which is closer to maternal perception. The duration and frequency of UC obtained from EHG and TOCO were not significantly different (p > 0.05). In conclusion, the proposed UC recognition algorithm has high accuracy and simple calculation which could be used for real-time analysis of EHG signals and long-term monitoring of UCs.

## Introduction

Uterine contraction (UC) is the result of the electrical activity within the myometrium. As labour approaches, UCs become more intense and synchronized, eventually expel fetus^1–3^. Therefore, UC monitoring provides essential clinical indicators in the progress of labour and delivery^4,5^, especially for women at the risk of preterm delivery.

Manual palpation, which identifies UC by palpating the parturient’s abdomen over the uterine, needs constant bedside presence which adds extra work for clinicians^6^. The pregnant women can feel UCs but may not record them in time. External tocodynamometry (TOCO) is the most widely used technique to monitor uterine activity during pregnancy and delivery^7^. However, TOCO is affected by the tightness of the belt and transducer position on the maternal abdomen^3^. Additionally, it is susceptible to motion artifacts and unreliable in obese women^8^. Intrauterine pressure catheter (IUPC) directly measures the intrauterine pressure changes caused by UCs but is limited by its invasiveness which can cause ruptured membranes and infection^9^.

Electrohysterogram (EHG) is a promising noninvasive technology considered to facilitate external uterine monitoring. With electrodes placed on the maternal abdomen, EHG can provide an objective evaluation of the myometrium activity by measurement of biopotentials underlying UCs^10^. EHG is more reliable and similar in accuracy to TOCO in detecting UCs compared to IUPC^11^ and is applicable for long-term monitoring uterine activity throughout pregnancy and delivery. The EHG signal can be modelled as an action potential fast wave whose amplitude is modulated by the slow wave corresponding to the frequency of the contractions. During pregnancy, EHG signals are characterized by a slow cyclic pattern of bursts of action potentials followed by a period of quiescence^12^. It has been reported that the bursts of action potential spikes occurred in both intrauterine and abdominal electrical signals synchronized with the UCs identified by simultaneously recorded intrauterine pressure^13^. The slow wave expressed by the envelope of burst has an essential meaning for the analysis of uterine activity, and its amplitude is affected by various conditions^13,14^. Therefore, EHG burst associated with UC could be detected, and its envelope of slow wave is described to replace the routine clinical TOCO for long-term ambulatory uterine monitoring.

Our team has explored the decision tree^15^ and the convolutional neural network^16^ to recognize EHG segments of UCs and non-UCs. However, to train these classifiers, EHG signals corresponding to UCs and non-UCs have to be segmented elaborately by manual referring to TOCO^17^. Moreover, a large number of EHG segments are required to improve the performance of these classifiers.

Nonlinear correlation analysis for burst extraction has been combined with fusion and elimination tests^18^ or Gaussian mixture models to detect UCs. These approaches allowed to detect a great majority of the contractions but also many artifacts. The false detection rate is also high, which could be due to the incomplete reference from manual segmentation^19^. Recently, unsupervised clustering method was applied to classify different types of UCs using complete spectrum projection of EHG signals^20^. Granger causal analysis of contraction and non-contraction was performed to extract EHG features. It was subsequently utilized by a quadratic discriminator classifier, which achieved high discriminatory power between term and preterm births^21^. A deep neural network was developed for semi-automatic identification of term and preterm uterine recording^22^. Although the good performance was achieved, manual segmentation contraction intervals and dummy intervals were required.

As a reliable and straightforward approach for on-line analysis, the root mean square (RMS) of the EHG signal within a sliding window was calculated to extract slow wave^23^. Besides, the method of higher-order zero crossings was proposed to discriminate between UC segments which relied upon the conclusion that the number of zero-crossings determined in a contraction segment significantly differs from a non-contraction segment^24^. The comparison study between EHG and TOCO showed that both methods demonstrate high agreement concerning the number of UCs recognized^13^. However, another paper reported that EHG was able to detect a higher number of UCs than TOCO identified by the experts with appropriate electrophysiological training^23^. Most studies compared their results with TOCO, which is not quite acceptable by obstetricians.

The duration and interval of UCs are not consistent at different gestational weeks (GWs) or even at the same recording. Automated detection of UCs is of great help for clinical evaluation and understanding the physiological activity of uterus during pregnancy. Most of the published papers focused on the use of pattern recognition techniques to extract features from EHG signals and evaluates various classifiers for detecting term and preterm delivery^21,22,26–28^. However, manual UC segmentation is usually required for reference, which affects the classification accuracy. Besides, more data from the various dataset are in demand to test and improve the current methods. We should also notice that on-line analysis limits the complexity of the automatic recognition algorithm^29^. Therefore, a fast and efficient algorithm is necessary to detect UCs with EHG signals in real-time.

In this paper, we propose a method for automatic recognition of UCs with EHG signals. ZCR related algorithm is used to highlight the EHG burst of UC, and its envelope is extracted to obtain TOCO-like signals for the convenience of clinicians. UCs from EHG signals are compared with maternal perception and TOCO. The number, duration, frequency and interval of UCs are provided for long-term monitoring of uterine activities and prediction of abnormalities in time.

## Results

### UCs recognition result

As shown in Table 1, 451 UCs were labelled with an average of 8 ~ 9 UCs for each pregnant woman during the 30-min recording. The number of UCs recognized by EHG is larger than TOCO, which is closer to maternal perception. Besides, the number of UCs that were correctly recognized (TP) by EHG is larger than TOCO. While the number of UCs and non-UCs that were falsely recognized (FP and FN) by EHG are smaller than TOCO. Both the sensitivity and PPV of EHG are higher than TOCO.

**Table 1:** UC recognition results.

	Number of UCs recognized	TP	FP	FN	Sensitivity (%)	PPV (%)
Maternal perception	451	N/A	N/A	N/A	N/A	N/A
EHG	425	396	29	55	87.80	93.18
TOCO	417	379	38	72	84.04	90.89

N/A: not available.

Table 2 shows the UC parameters obtained from EHG and TOCO. No significant difference was found in the UC parameters between EHG and TOCO (p > 0.05).

**Table 2:** UC parameters obtained from EHG and TOCO.

UC parameter	EHG (n = 54)	TOCO (n = 54)
Number of UC (times)	425	417
Frequency of UC (times/10 min)	3.162 ± 0.926	3.149 ± 0.856
Duration of UC (min)	0.619 ± 0.238	0.624 ± 0.160
Interval of UC (min)	3.145 ± 1.088	3.270 ± 1.756

### Results of preterm and term delivery

Table 3 shows the UC parameters of term and preterm delivery obtained from EHG and TOCO signals. No significant difference was found in the UC parameters between preterm and term delivery (p > 0.05), whether by EHG or by TOCO.

**Table 3:** UC parameters from preterm and term delivery.

UC parameter	Preterm delivery (n = 4)	Term delivery (n = 50)
Number of UC from EHG (times)	26	399
Frequency of UC from EHG (times/10 min)	3.253 ± 0.862	3.155 ± 0.931
Duration of UC from EHG (min)	0.783 ± 0.469	0.609 ± 0.210
Interval of UC from EHG (min)	3.234 ± 0.933	3.138 ± 1.100
Number of UC from TOCO (times)	26	391
Frequency of UC from TOCO (times/10 min)	3.358 ± 0.574	3.132 ± 0.873
Duration of UC from TOCO (min)	0.610 ± 0.125	0.625 ± 0.162
Interval of UC from TOCO (min)	4.916 ± 4.077	3.139 ± 1.329

### The offset coefficient α

**Figure 1:**
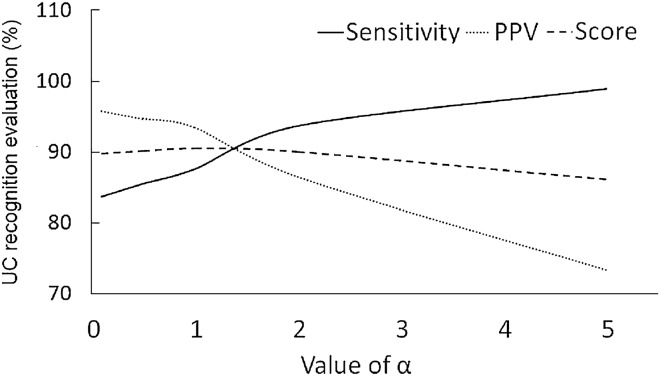
The influence of $\alpha$ on the recognition result.

The offset coefficient α determined the degree to which the EHG signal was elevated, and therefore influenced the subsequent ZCR. As shown in Fig. 1, the sensitivity of UC recognition increases with α while PPV decreases with α. The score is the average of sensitivity and PPV. With the comprehensive consideration of the changes of sensitivity, PPV and score, we selected α between [1, 2].

### The exponent γ of ZCR power function

**Figure 2:**
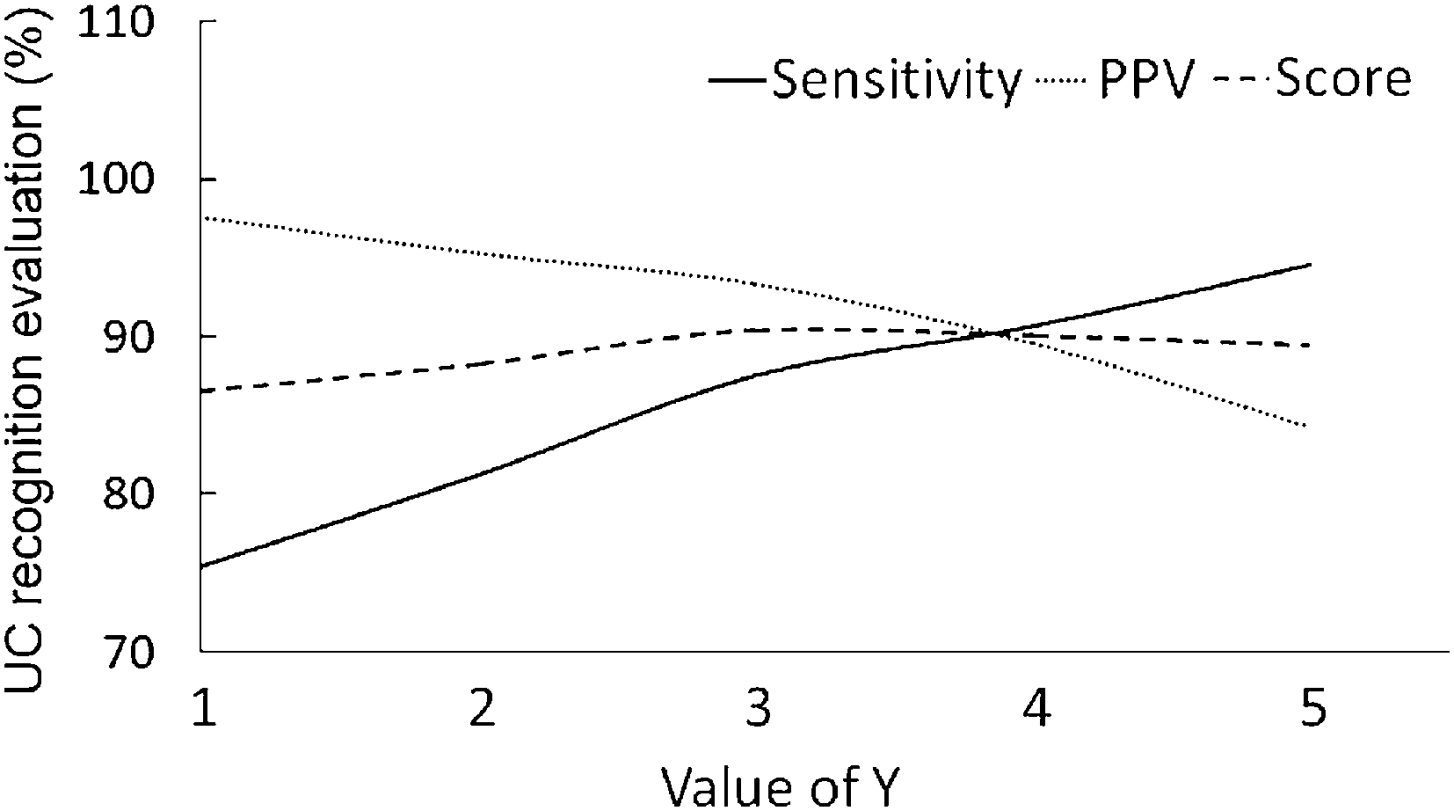
The influence of γ on the recognition result.

The exponent γ affected the ratio of UC to non-UC and the subsequent UC recognition. As shown in Fig. 2, the sensitivity of UC recognition increases with γ while PPV decreases with γ. The score is the average of sensitivity and PPV. With the comprehensive consideration of the changes of sensitivity, PPV and score, we selected γ between [3, 4].

## Discussion

UCs are routinely and constantly monitored during pregnancy and delivery. The amplitude and frequency of UCs varied widely during the gestation period, and thus quantitative assessment of UCs can guide obstetricians to choose appropriate therapies. EHG signals have been investigated to monitor uterine activities in recent years. However, EHG signals are usually manually segmented for identifying UCs. This paper proposed a novel algorithm for automatic recognition of UCs from EHG signals without manual segmentation or referring to TOCO signal. With easy and fast on-line analysis, it provides TOCO-like signals acceptable to obstetricians and could be employed to monitor UCs clinically.

The number of zero crossings has been shown to be related to the frequency content of the EHG signal, and the zero-crossings determined in UC segments significantly differ from the non-UCs^30^. However, zero-crossing-based techniques have not been further validated in subsequent studies. In this paper, ZCR was extracted to represent the variation of the EHG signal or the frequency content. Considering the amplitude information, we utilized the power of ZCR to modulate the EHG signal to reinforce the difference between the segments of UC and non-UC. Then, the envelope of EHG burst was achieved to recognize UC, and consequently, the parameters of UC were obtained.

We noticed most of the studies on UC detection with EHG signals compared their results with IUPC or TOCO^18,30^. Although IUPC can provide accurate information of UCs, it is seldom applied to normal pregnant women in the clinical practice, and therefore the data from IUPC is quite limited. TOCO is more susceptible to the interference from maternal movement or shifting of the abdominal transducer, particularly unreliable in obese parturients. Maternal perception can also indicate UCs, which is first used as a reference in this study. Mostly, pregnant women can feel abdominal pain and tightness when UCs are coming. Even though not all UCs can be labelled by the pregnant women due to the increasing pain during the recording period, at least all the UCs labelled by the pregnant women are convincing and therefore could be taken as an evaluation criterion for UC recognition. To the best of our knowledge, only our team has investigated the time difference between the onset of UCs determined from TOCO and maternal perception^31^. The maternal perception can suggest a UC but not the onset, the peak or the end of a UC. Therefore, a UC identified by EHG burst or TOCO within a time interval to maternal perception could be regarded as a true UC. Once a UC was determined, its number, duration, interval and frequency could be obtained, which are indispensable parameters for clinical monitoring.

The proposed algorithm in this paper shows higher sensitivity and PPV in detecting UC than TOCO, the convention method of UC detection. The number of UCs detected with EHG is closer to maternal perception. Also, the published papers reported EHG presents higher consistency to IUPC than TOCO for monitoring uterine activities^9,32^. The UC parameters in this paper are similar to the published results^6,13^ and are clinically reasonable. Besides, they are not significantly different between EHG and TOCO. For automatic UC detection, our results achieved by simple calculations are comparable to the algorithm of nonlinear correlation coefficient^18^.

UC parameters from both preterm and term delivery obtained by EHG and TOCO were reported in this study. However, UC parameters were not significantly different between preterm and term delivery, which could be due to fewer EHG recording from preterm delivery.

In a further study, more clinical data from various GWs and preterm delivery have to be collected to validate the proposed algorithm. Maternal perception has to be confirmed by obstetrician's palpation at the bedside to annotate UCs thoroughly.

In summary, with the advantage of high accuracy and simple calculation, the ZCR-based automatic UC recognition algorithm with the EHG signal is convincing. It has tremendous application potential for real-time and long- term UC monitoring.

## Methods

**Figure 3:**
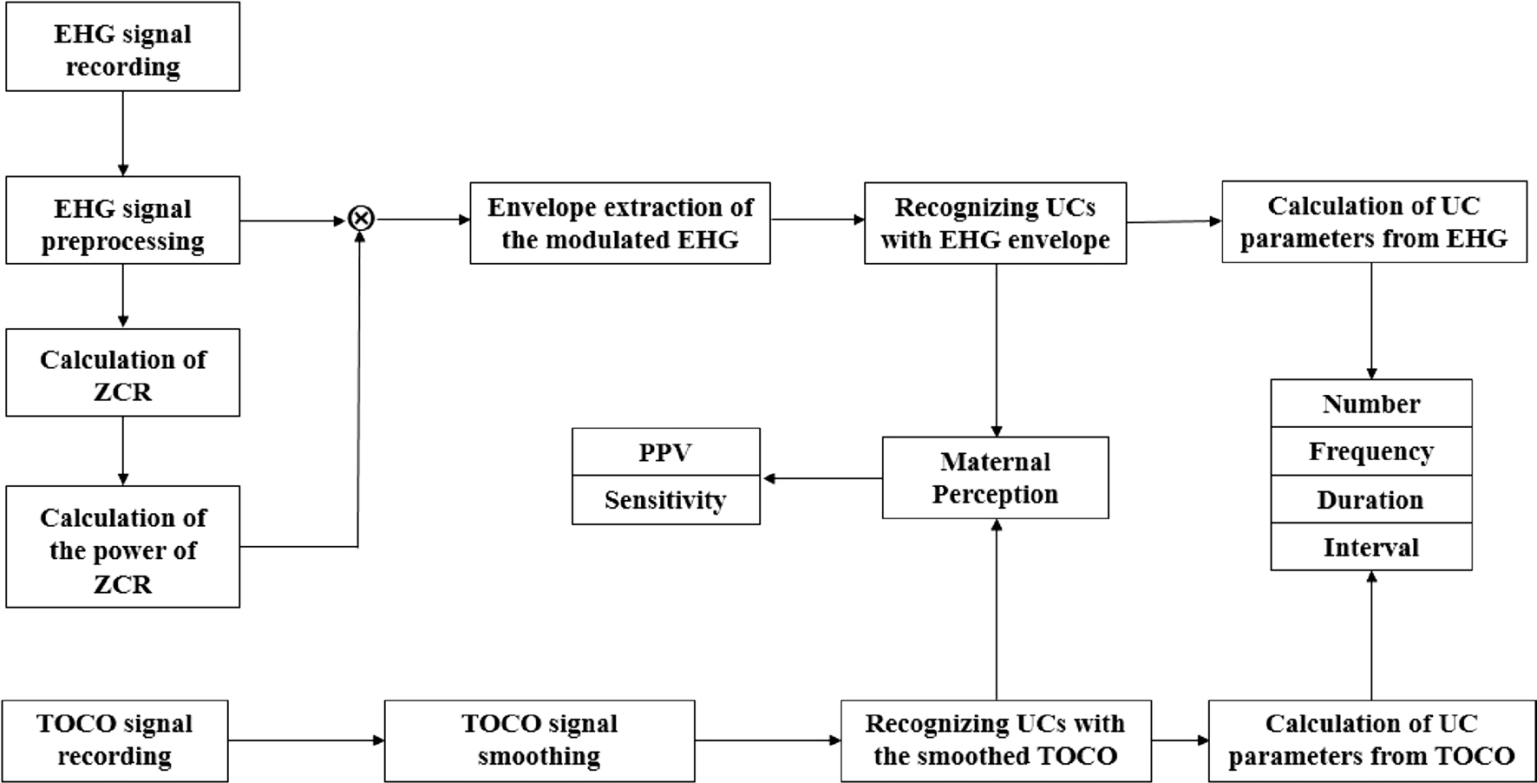
Block diagram of recognition and evaluation of UCs with EHG and TOCO.

As shown in Fig. 3, the block diagram of automatic UC recognition includes EHG signal recording, EHG signal preprocessing, calculation of zero-cross rate (ZCR), calculation of the power of ZCR and envelope extraction of the modulated EHG. UCs were recognized by the modulated EHG and TOCO signals, respectively, and compared with maternal perception in terms of positive predictive value (PPV) and sensitivity. UC parameters, including number, frequency, duration and interval, were obtained from EHG and TOCO signals.

### EHG recording

As shown in Table 4, a total of 54 pregnant women with 33 to 41 GWs were recruited in Beijing Union Medical College Hospital in China. Four of them were preterm delivery (delivered before 37 GWs), and the others were term delivery (delivered after 37 GWs). The age, height, weight, average GWs, minimum GWs and maximum GWs of preterm and term pregnant women were given in Table 4. The study was approved by the Local Ethics Committee of Beijing Union Medical College Hospital and was conducted strictly according to the Declaration of Helsinki (1989) of the World Medical Association. The pregnant women were asked to sign consent after being informed of the aims, potential benefits and risks of the study.

**Table 4:** Basic information of pregnant women.

Delivery outcome	Preterm delivery	Term delivery
Number of pregnant women	4	50
Age (years/Mean ± SD)	30 ± 3	31 ± 4
Height (cm/Mean ± SD)	162 ± 3	164 ± 4
Weight (kg/Mean ± SD)	75 ± 5	73 ± 9
Mean GWs (weeks + days)	35 + 2	40 + 2
Minimum GWs(weeks + days)	33 + 2	38 + 0
Maximum GWs(weeks + days)	36 + 6	41 + 1

SD: standard deviation.

**Figure 4:**
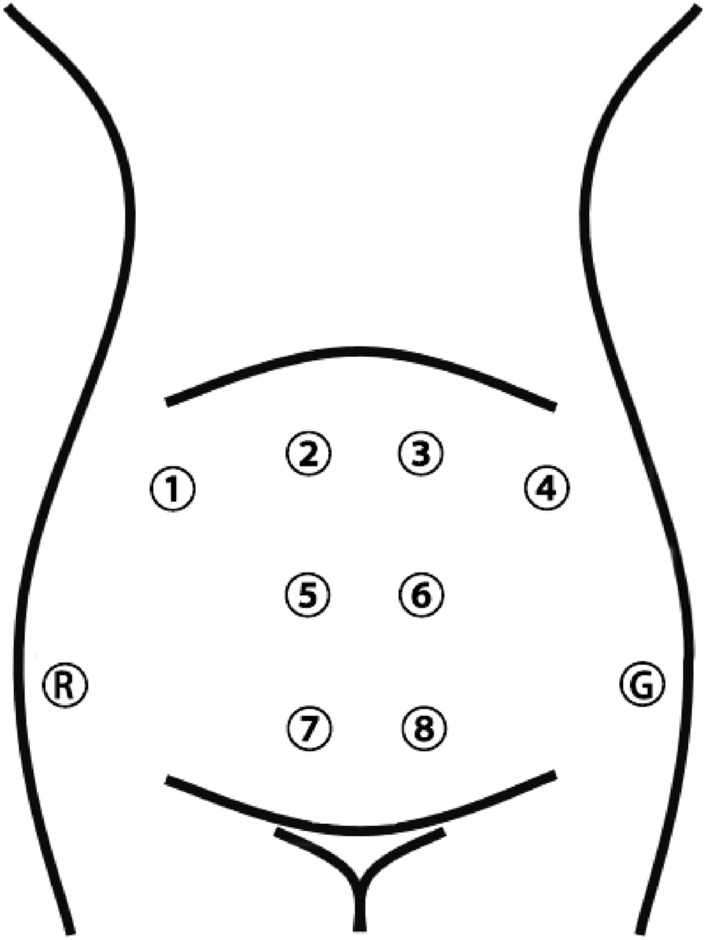
The configuration of the 8-electrodes on the abdomen.

Eight-channel EHG signals and a TOCO signal were recorded simultaneously using a bespoke device in our lab. As shown in Fig. 4, electrodes 1 to 4 were placed on the fundus of the uterus, electrodes 5 and 6 symmetrically placed below the navel, electrodes 7 and 8 were placed on the uterine cervix, and the reference (R) and ground (G) electrodes were placed on the left and right ilium. The EHG and TOCO signals were sampled at 250 Hz for approximately 30 min. The pregnant woman pressed a button to label a UC she felt during the signal recording. Meanwhile, a researcher has been staying at the bedside to write UCs in a notebook during the recording. The UCs labelled by both of them were adopted for analysis.

### EHG signal preprocessing

The 8-electrode EHG signals were averaged and then preprocessed by a 4th-order Butterworth low-pass filter with the cut off frequency of 3 Hz, and then a median filter to remove the unwanted signals from maternal electrocardiograph, respiration, movement and power line interference.

### Zero-cross rate of EHG signal

Each burst of EHG signal has been known to be a period of elevated electrical activity associated with a UC^14^. The amplitude of the EHG signal is often influenced by electrode placement, GWs, as well as the individual difference. Therefore, the EHG amplitude and power spectral cannot be applied directly to evaluate UCs from different pregnant women. Only the variation of amplitude-based parameters is valuable in clinical application^13^.

ZCR is the rate of sign-changes at which the signal changes from positive to zero to negative or from negative to zero to positive^33^. ZCR is often used to extract the signal feature and has some relationship with frequency, which is useful in analyzing various physiological signals^25,33^.

In this paper, ZCR was obtained by elevation of EHG amplitude and calculation in a sliding window.

### Elevation of EHG signal

The EHG signal was elevated αE to highlight the EHG burst and reduce the impact of the non-UC segment. α is the offset coefficient and E is the mean of EHG amplitude. E was calculated by the formula (1).

1 $$ {\text{E}} = \frac{{\sum\nolimits_{i = 0}^{n - 1} {x(i)} }}{n} $$

where *x*(*i*) represents the EHG amplitude at the point *i*, n represents the length of the EHG signal in point.

The offset coefficient $\upalpha  \in \left[ {0, \, 5} \right]$ was selected in this study, which was the tradeoff between retaining useful information and reducing interference.

### Calculation of zero-crossing rate within a sliding window

A sliding window was applied to the elevated EHG signal with the moving step of 1 sampling point. ZCR within the window was calculated by the formula (2).

2 $$ {\text{Z}} = \frac{m}{W} \times 100\% $$

where m is the number of zero-crossing points within the sliding window, and W is the length of the window.

**Figure 5:**
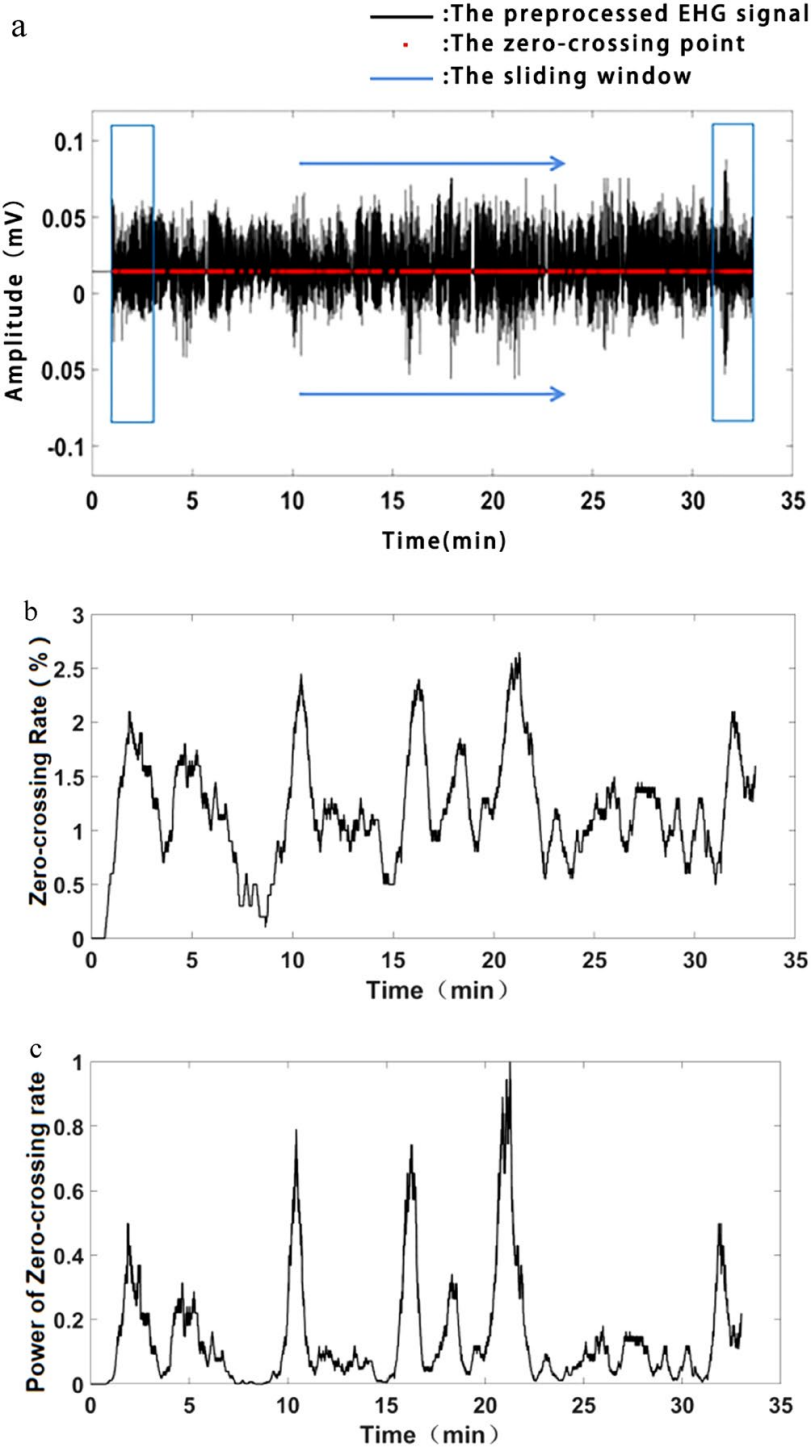
Strengthening of EHG burst. (**a**) A sliding window moving along the elevated EHG signal. The zero-crossing points are in red, and the sliding window is in blue. (**b**) The zero-crossing rate. (**c**) Power of zero-crossing rate.

Generally, a UC duration of 30–60 s was reported in the previous research^25^. Here, the length of the sliding window was set to 40 s to cover an EHG segment of UC. With the sampling rate of 250 Hz, the length of the sliding window was W = 10,000 points. Figure 5 shows the zero-crossing points in red and the sliding window in blue. Figure 5 b shows the ZCR curve, which the higher ZCR, the more uterine activity.

### Power of the zero-crossing rate

The ZCR value was normalized to [0, 1] to overcome individual differences. Then, γth (γ > 1) power of ZCR or ZCR^γ^ was calculated to strengthen the larger ZCR and attenuate the smaller ZCR, see Fig. 5c. The power of ZCR was applied to modulate the preprocessed EHG signal to improve the ratio of UC to non-UC segments.

### Recognition of UCs with the modulated EHG

**Figure 6:**
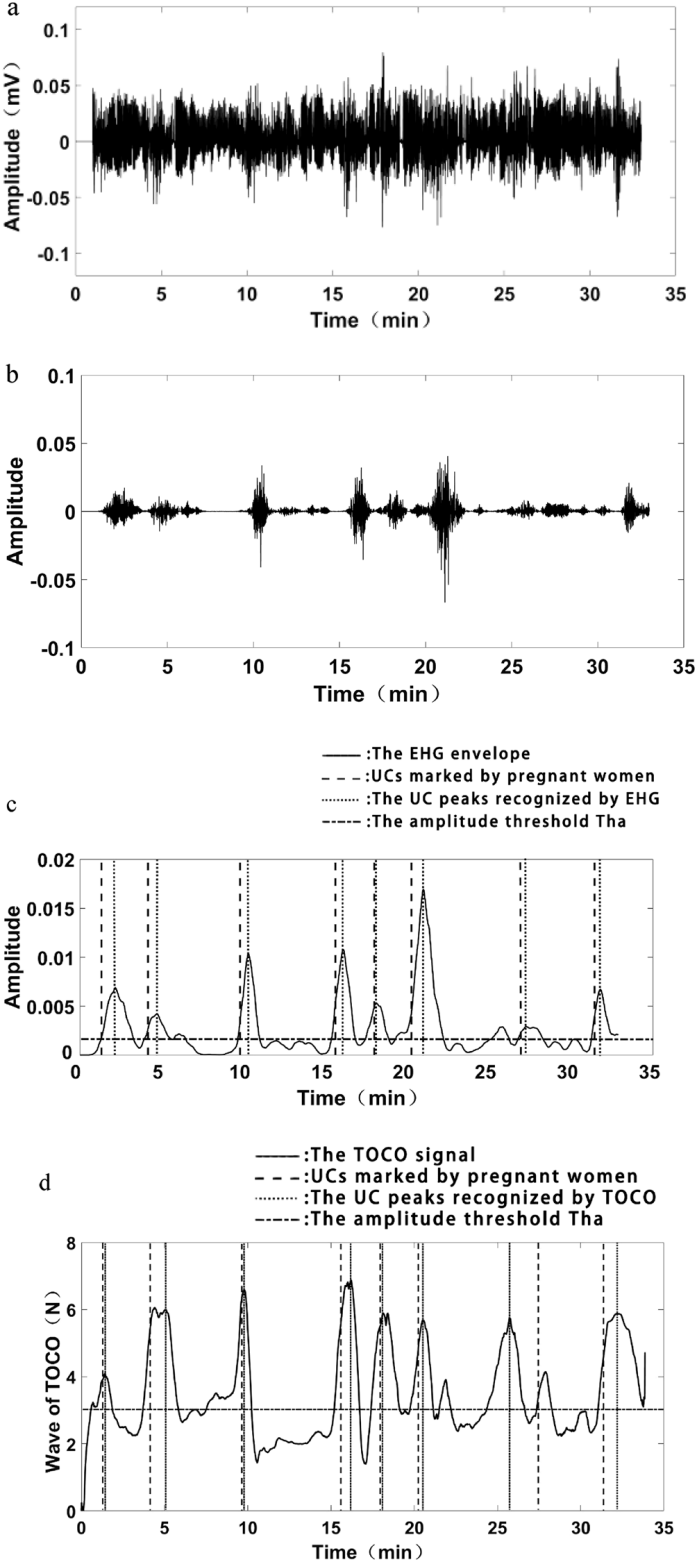
Recognition of UCs with EHG and TOCO. (**a**) Preprocessed EHG signal. (**b**) The modulated EHG signal. (**c**) Recognition of UCs with EHG envelope. (**d**) Recognition of UCs with TOCO signal.

As shown in Fig. 6a,b, the preprocessed EHG signal was modulated by ZCR^γ^ to strength the bursts corresponding to UCs, and attenuate the EHG segments corresponding to non-UCs. Then the envelope of the modulated EHG signal was obtained by RMS, which is simple and applicable for on-line analysis. RMS was calculated by the formula (3).

3 $$ {\text{RMS}} = \sqrt {\frac{1}{N}\sum\limits_{i = 0}^{N - 1} {x(i)^{2} } } $$

where *x*(*i*) represents the amplitude of the modulated EHG signal at the point *i*, N stands for the length of 10 s EHG segment, here N = 2500. In this study, when the window length was 10 s (N = 2500), the envelope waveform obtained was the most similar to EHG signal.

A UC was identified if its amplitude was larger than the threshold Tha and its duration was larger than the threshold Thd, where Tha is the mean amplitude of EHG envelop, and Thd is 30 s in terms of UC duration. As shown in Fig. 6c, a UC was correctly identified if its peak position in dotted line was within 20 s interval to the maternal perception in the dash line.

### Recognition of UCs with TOCO

As shown in Fig. 6d, TOCO wave was smoothed by averaging multiple points in 40 s, then a UC was recognized with a similar method as EHG envelope.

### Evaluation of UC recognition

In this paper, we assumed that the UCs labelled by maternal perception are reliable. All of the UCs obtained from EHG and TOCO were evaluated by their intervals to maternal perception. Only sensitivity and PPV were applied to evaluate the UC recognition results because the pregnant woman labelled not all UCs due to the acute pain during signal recording. The sensitivity and PPV were calculated by the formula (4) and (5):

4 $$ Sensitive = \frac{TP}{{TP + FN}} $$

5 $$ PPV = \frac{TP}{{TP + FP}} $$

where TP (true positive) represents the UCs correctly recognized by EHG or TOCO in terms of maternal perception. FP (false positive) represents the UCs recognized by EHG or TOCO but not labelled by maternal perception. FN (false negative) represents the UCs not recognized by EHG or TOCO but labelled by maternal perception.

### Calculation of UC parameters

**Figure 7:**
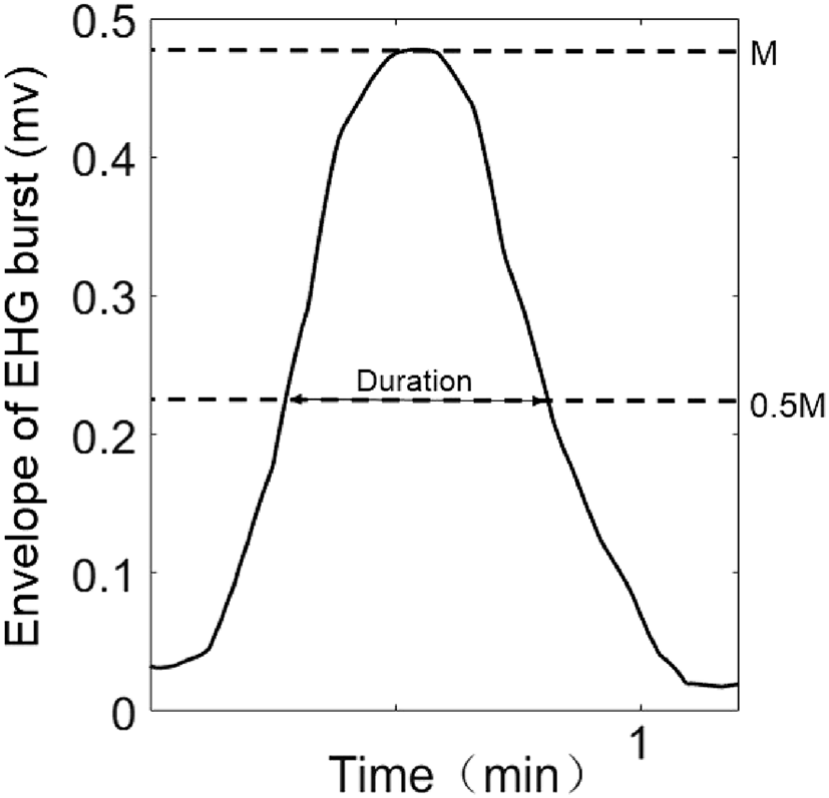
The definition of UC duration.

The number of UCs during the recording period and within 10 min were counted respectively. The interval of UCs was defined as the time interval between the adjacent UC peaks. The duration of UCs was defined as the half-wave width of the envelope, as shown in Fig. 7.

### Statistical analysis

The non-parametric t-test (Mann–Whitney U test) was performed using SPSS 22 (IBM Corporation, New York, United States) to assess the difference of UC parameters between EHG and TOCO, and between preterm and term delivery. P < 0.05 was considered statistically significant.

### Ethical approval

The study was approved by the Local Ethics Committee of Beijing Union Medical College Hospital and was conducted strictly according to the Declaration of Helsinki (1989) of the World Medical Association. The pregnant women were asked to sign consent after being informed of the aims, potential benefits and risks of the study. This article does not contain any studies with animals performed by any of the authors.

### Informed consent

Informed consent was obtained from all individual participants included in the study.
